# RdsA Is a Global Regulator That Controls Cell Shape and Division in *Rhizobium etli*

**DOI:** 10.3389/fmicb.2022.858440

**Published:** 2022-04-07

**Authors:** Sofía Martínez-Absalón, Carmen Guadarrama, Araceli Dávalos, David Romero

**Affiliations:** Programa de Ingeniería Genómica, Centro de Ciencias Genómicas, Universidad Nacional Autónoma de México, Cuernavaca, Mexico

**Keywords:** two-component systems, peptidoglycan synthesis, polar growth, bacterial divisome, gene knockdown

## Abstract

Unlike other bacteria, cell growth in rhizobiales is unipolar and asymmetric. The regulation of cell division, and its coordination with metabolic processes is an active field of research. In *Rhizobium etli*, gene RHE_PE00024, located in a secondary chromosome, is essential for growth. This gene encodes a predicted hybrid histidine kinase sensor protein, participating in a, as yet undescribed, two-component signaling system. In this work, we show that a conditional knockdown mutant (cKD24) in RHE_PE00024 (hereby referred as *rdsA*, after rhizobium division and shape) generates a striking phenotype, where nearly 64% of the cells present a round shape, with stochastic and uncoordinated cell division. For rod-shaped cells, a large fraction (12 to 29%, depending on their origin) present growth from the old pole, a sector that is normally inactive for growth in a wild-type cell. A fraction of the cells (1 to 3%) showed also multiple ectopic polar growths. Homodimerization of RdsA appears to be required for normal function. RNAseq analysis of mutant cKD24 reveals global changes, with downregulated genes in at least five biological processes: cell division, wall biogenesis, respiration, translation, and motility. These modifications may affect proper structuring of the divisome, as well as peptidoglycan synthesis. Together, these results indicate that the hybrid histidine kinase RdsA is an essential global regulator influencing cell division and cell shape in *R. etli*.

## Introduction

In bacteria, there is a tight relationship between cell shape and cell division. Cell shape is ultimately determined by the cell membrane and the cell wall (Cava et al., 2013; Kysela et al., 2016). For rod-shaped (bacillary) cells such as *Escherichia coli*, cell growth occurs by dispersed incorporation of new peptidoglycan (PG) alongside the lateral wall, followed by PG synthesis at the midpoint of the cell, where the septum will be located at cell division (Dewachter et al., 2018). Studies in *E. coli* of the cell machinery needed for growth and division and their control has revealed an astonishing level of complexity (Männik and Bailey, 2015; Haeusser and Margolin, 2016; Dewachter et al., 2018; Krupka and Margolin, 2018; Levin and Janakiraman, 2021; Straume et al., 2021). These insights have been expanded, both in scope and complexity, by the study of new bacterial groups.

In α-proteobacteria, cell cycle regulation is closely related to their lifestyle. *Caulobacter crescentus*, unlike other bacteria, generates upon division two cells with different morphology and physiology. The mother cell is sessile and active for cell division. The daughter cell is a mobile cell, inactive for cell division and replication (Laub et al., 2002). Asymmetrical cell division is a widespread characteristic among α-proteobacteria (Hallez et al., 2004). Understanding the mechanisms that lead to the formation of these specialized cells and the coordination of cell division has motivated an intense research (Curtis and Brun, 2010; Collier, 2016; Woldemeskel and Goley, 2017). Moreover, it has revealed the presence of a complex regulatory mechanism, with a hierarchical cascade of transcriptional regulators, in which the CtrA regulator has a central role in the coordination of cell division (Quon et al., 1996; Laub et al., 2000, 2002; Mann et al., 2016; Delaby et al., 2019). CtrA controls, either directly or indirectly, 26% of the cell cycle-regulated genes in *C. crescentus* (Laub et al., 2000).

Two-component regulatory systems are of paramount importance to achieve control of *C. crescentus* cell-cycle regulated genes. When the hybrid histidine kinase sensor protein CckA is phosphorylated, it can transfer the phosphate group to the phosphotransfer protein ChpT, which in turn phosphorylate the response regulator CtrA. The phosphorylated form of CtrA is responsible for transcriptional activation of its target genes. The kinase activity of CckA is shut off by the action of the phosphorylated form of the response regulator DivK, stabilized by the atypical histidine kinase DivL. Phosphorylation of DivK is controlled by the histidine kinase DivJ and the phosphatase PleC. PleC also modulates phosphorylation of the diguanylate cyclase PleD, leading to the synthesis of cyclic di-GMP. Switching of CckA from a kinase to a phosphatase activity is instrumented by binding of cyclic di-GMP, effectively precluding phosphorylation of CtrA and activation of their target genes (see Poncin et al., 2018 and references therein).

Although the general architecture for regulation of cell division is conserved among the α-proteobacteria, there are also interesting departures from this scheme. Bacteria of the order Rhizobiales lack various proteins (MreB MreCD, PBP2, RodA, and RodZ) of the elongasome complex (Egan et al., 2020). Rhizobiales with bacillary morphology, such as *Sinorhizobium meliloti* and *Agrobacterium tumefaciens*, carry out cell growth in a distinctive manner. In both bacteria, cell growth is asymmetric and polarized, but cell elongation occurs through the polymerization of PG at a single cell pole, called the new pole. The opposite pole, called the old pole, remains inactive for polymerization (Brown et al., 2012; Cameron et al., 2015). Further rounds of cell division maintain both the asymmetry and polarization, growing exclusively from the new cell poles. How unipolar growth takes place and the identification of the mechanisms allowing the distinction between the old and new poles in Rhizobiales is an active field of research. Transcriptional regulation of the cell division cycle has also revealed new variations. Although the centrality of the CtrA regulator is maintained, the transcriptional network reveals some plasticity, where some modules are maintained but other new modules are integrated into the network among the different bacteria that constitute the group (Pini et al., 2015; Poncin et al., 2018).

The rod-shaped bacterium *Rhizobium etli* is a nitrogen-fixing bean symbiont, widely studied for its use as a biofertilizer and interesting genome architecture (González et al., 2006). The *R. etli* CFN42 genome consists of six extra-chromosomal replicons (ranging in size from 184 to 642 kb) and one chromosome. Five of these extra-chromosomal replicons (plasmids p42a, p4b, p42c, p42d, and p42f) are dispensable for growth under laboratory conditions in rich medium (Brom et al., 1992, 2000). Plasmid p42e (505 kb) cannot be eliminated from the cell, appearing to be a secondary chromosome. Nearly 10% of the gene content in p42e participates in central metabolic processes, but it also contains two essential genes (Landeta et al., 2011). Genes RHE_PE00001 and RHE_PE00024 were proposed as essential genes because their mutation is lethal for the cell. Convincing orthologs for both genes are mainly restricted to the Rhizobiales, including Agrobacterium and Rhizobium species. Gene RHE_PE00001 codes for a conserved hypothetical protein, consisting of a Domain of Unknown Function (DUF1612) conserved among the Rhizobiales, and a helix-turn-helix DNA binding motif in the N-terminus. Gene RHE_PE00024 encodes a predicted hybrid histidine kinase (HK) sensor protein, that is part of a two-component regulatory system of as yet unknown function (Landeta et al., 2011).

In this work, we unravel the function of the hybrid histidine kinase sensor protein encoded in RHE_PE00024, by studying the phenotypic effects displayed by a conditional knockdown mutant (cKD24) in this gene. Depletion in the RHE_PE00024 encoded protein generated a striking change in cell morphology, characterized by the finding of a high proportion (nearly 64%) of round, nearly spherical cells. Both the round and bacillary cells present a lethargic, stochastic and uncoordinated cell division. A significant proportion of bacillary cells (12 to 29%, depending on their origin) display growth from the old pole, a sector that is normally inactive for growth in a wild-type cell. Branched cells, the product of ectopic growth poles that do not complete division, were also seen. RNAseq analysis of the cKD24 mutant showed that the predicted hybrid histidine kinase sensor protein encoded by RHE_PE00024 acts as a global regulator, controlling, either directly or indirectly, genes participating in five different biological processes (cell division, PG synthesis, oxidative respiration, translation and motility). Based on these results, we propose to name this gene *rdsA* (after *r*hizobium *d*ivision and *s*hape).

## Results

### Change in Cell Shape in a Conditional Knockdown Mutant for *rdsA*

**FIGURE 1:**
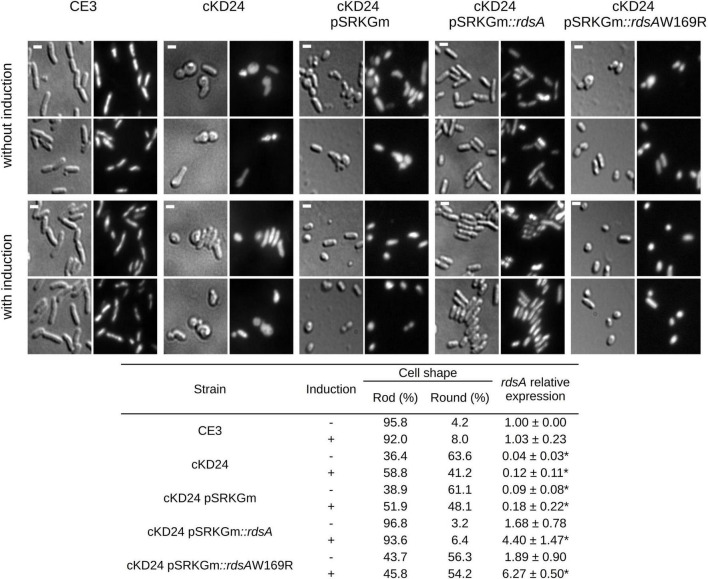
Depletion of RdsA causes a change in cell shape. The top part shows the microscopic analysis of uninduced (top two rows) or induced cells (bottom two rows) for different strains. CE3 (*R. etli* wild type), RdsA depleted strains (cKD24, cKD24 pSRKGm) or complemented strains (cKD24 pSRKGm*:rdsA*, cKD24 pSRKGm*:rdsA*W169R). After growth in liquid PY media for 10 h without or with induction, cells were spotted on a 1% agar pad. Each light background image (DIC images) has a corresponding image using DAPI staining (dark background). Scale bar, 1.5 μm. The table at the bottom of the figure shows the percentage of cells with a rod or round shape. Percentages were obtained by counting at least 900 cells for each strain/condition through three independent biological replicas. The table also shows *rdsA* relative expression obtained by RT-qPCR, after normalization with the *rpoA* constitutive gene. Data are means (± SD) obtained from three independent biological replicates (each with three experimental replicates) for every treatment. Statistically significant differences between *R. etli* CE3 RQ and the five cKD24 mutant strain RQ levels are marked with asterisks, and were evaluated with a Student’s *t*-test (*P* < 0.05).

Gene *rdsA* (RHE_PE00024) encodes the sensory component of an essential hybrid Histidine Kinase (HK) regulatory system. Canonical HK proteins constitute the sensor part of two-component regulatory systems. In these, the canonical HK contains an input domain able to sense regulatory stimuli and subsequently the catalytic ATP binding (CA) domain phosphorylates a His residue in the dimeric histidine phosphotransfer (DHp) domain. The phosphoryl group is then transferred to an Asp residue in the receiver (REC) domain of a separate response regulator protein (Gao and Stock, 2009; Rodríguez et al., 2020). In contrast, the predicted hybrid histidine kinase RHE_PE00024 is comprised of a putative N-terminal sensory region (containing two PAS sensor domains) and a kinase core containing both the CA and the DHp domains. Additionally, it contains a REC domain with a potentially phosphorylable Asp residue. In systems of this kind, transfer of the phosphoryl group to the corresponding response regulator protein frequently entails an intermediate protein with a phosphorylable His residue, designated as histidine phosphotransferase or Hpt (Gao and Stock, 2009). No genes encoding a plausible response element or a Hpt protein were found in its vicinity. To better understand the possible function of RdsA, we implemented a conditional knockdown system in which the *rdsA* gene is put under the control of a cumate inducible promoter (Chubiz et al., 2013). In the corresponding mutant (cKD24), expression of *rdsA* depends on the inducible cumate system (see section “Materials and Methods”). Interestingly, growth of the cKD24 mutant in rich medium without cumate was slower [growth rate constant (μ) = 0.23 h^–1^, mean generation time (g) = 2.9 h] than the wild type cells (μ = 0.33 h^–1^, g = 2 h, Supplementary Figure 1). The slower growth of cKD24 was observed even upon addition of cumate to the medium (μ = 0.22 h^–1^, g = 3.1 h). No significant differences were observed in relative viability between strains (data not shown). Notably, microscopical analysis of the cKD24 knockdown mutant showed a striking change in shape compared to the wild-type cells. In the absence of cumate, cKD24 showed 63.6% of cells with a round shape, while 36.4% maintained a bacillary shape (Figure 1). DAPI staining revealed nucleoid material in both kinds of cells (Figure 1), suggesting that they are viable. In contrast, when expression of *rdsA* is increased by addition of cumate, the cKD24 mutant showed a reduced proportion of round cells (41.2%), and a correspondingly higher proportion of bacillary cells (58.8%, Figure 1). Even in the presence of induction there are a high percentage of cells with a round shape, suggesting that wild-type expression levels of *rdsA* were not achieved. To verify this, *rdsA* gene expression was monitored by RT-qPCR analysis. In the absence of induction, a low expression level of *rdsA* was seen, at a level representing just 4.6% of the wild-type levels. However, for unknown reasons, cumate addition did not lead to high expression levels of the gene, showing only 12% of the wild-type levels (Figure 1). To enhance the level of expression of *rdsA* in the cKD24 mutant, we introduced supernumerary copies of the *rdsA* gene (under the control of an IPTG inducible system), in a replicative plasmid. The complemented strain (cKD24 pSRKGm*:rdsA*) showed expression levels of *rdsA* 1.68-fold higher than in the wild-type cells, even in the absence of induction; in contrast, a fourfold increase in expression compared to the wild-type was seen upon IPTG addition (Figure 1). Microscopical analysis of cells in the complemented cKD24 pSRKGm*:rdsA* strain, revealed a wild-type phenotype, where 97% of the cells showed a bacillary shape (Figure 1), with a low proportion of cells with a round shape. As a control, a mock complementation experiment was done, introducing an empty plasmid in mutant cKD24. Both the expression levels of *rdsA* (Figure 1) and proportion of round cells (61.1 and 48.1% without and with induction, respectively, Figure 1) were indistinguishable from the ones seen in the cKD24 mutant. Together, these results indicate that RdsA depletion causes an anomalous round shape in *R. etli* CFN42. Since cell morphology in Gram-negative bacteria is determined by a proper structuring of the cell wall (Cava et al., 2013), *rdsA* may participate in the regulation of cell wall biogenesis.

### Homodimerization Is Essential for Proper Functioning of the RdsA Hybrid Histidine Kinase

Many HK proteins require homodimerization to carry out their phosphorylation function (Mann et al., 2016). To evaluate if the HK-hybrid protein RdsA requires homodimerization for its function, an *Escherichia coli*, LexA-based two-hybrid system (Dmitrova et al., 1998) was used (Supplementary Figure 2). In this system, repression of a target reporter gene is seen only if a fusion of LexA with the desired protein is able to dimerize (Dmitrova et al., 1998). Most of the clones obtained with *rdsA* revealed a strong repression, indicating the ability of the corresponding protein to dimerize. Interestingly, one of the clones revealed a reduced ability to repress the target gene, indicating a reduced dimerization (Supplementary Figure 2). Although the amplification protocol used to generate these clones was not designed to introduce mutations, this clone displayed a spontaneous single-base change provoking an aminoacid change (W169R), located in the second PAS domain of RdsA. PAS domains are frequently involved in protein-protein interactions (Möglich et al., 2009). To evaluate if this failure to dimerize affects function, the mutant version *rdsA*W169R was introduced in a replicative plasmid (see section “Materials and Methods”). The cKD24 pSRKGm*:rdsA*W169R strain, showed 56 and 54% of round cells both in the absence and presence of induction, respectively (Figure 1), indicating a failure to complement the changes in shape characteristic of the cKD24 mutant. Failure for proper complementation was not due to an absence of expression of the mutant gene, as evaluated by a RT-qPCR analysis (Figure 1). These results are consistent with the interpretation that homodimerization of RdsA is essential for its function.

### RdsA Depletion Causes Uncoordinated and Stochastic Cell Division and Change of Polarity

**FIGURE 2:**
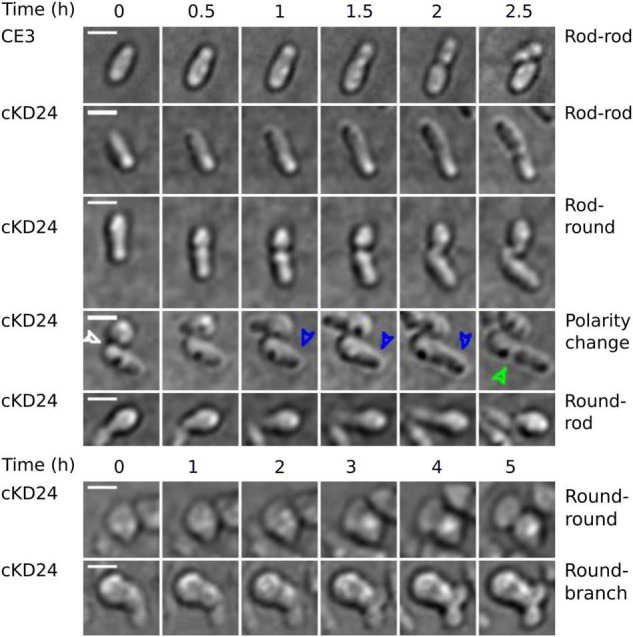
Depletion of RdsA provokes changes in cell division. Each row shows representative sequential images of time-lapse microscopical analysis of *R. etli* CE3 and cKD24 in absence of induction. Cells were grown in PY medium for 9 h and then spotted on agarose pads (1% in MMY medium). Cells were imaged every 10 min using DIC microscopy. For each block, time is shown at the top. On the right part, the type of change detected is indicated. In each row, the zero time corresponds to the beginning of the time-lapse experiment except for the cKD24 rows: polarity change, round-rod, and round-round where the zero time corresponds to cells imaged after 1:20 h, 30 min, and 2:30 h of growth, respectively. Recording times were chosen to optimize detection of each phenotype. In the row corresponding to polarity change in the cKD24 mutant, the first photograph shows the end of the cell division in a round cell producing a rod cell. This allows us to locate the new pole in the rod-shaped cell (white arrow). The rod cell elongates through the old pole (blue arrow), which is usually inactivated for growth in wild-type cells, finally, the cell septation begins (green arrow). Scale bars, 2 μm.

**FIGURE 3:**
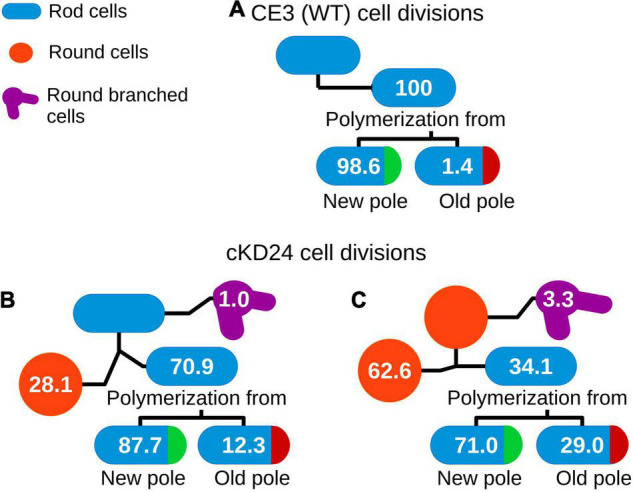
Cell fate in *R. etli* CE3 **(A)** and cKD24 mutant **(B,C)**. Tracking of cell division events for 10 h of growth on agarose pads (1% in MMY medium) in absence of induction. Cells were imaged each 10 min using DIC microscopy. Numbers inside of each cartoon cell represents the percentage of successive division events for each cell shape. N was 217 for CE3 cells, 103 for cKD24 bacillary cells, and 91 for cKD24 round cells.

To evaluate if depletion of RdsA lead to changes in cell division pattern, a time-lapse analysis of living cells was carried out (see section “Materials and Methods”). For this, the division pattern of both *R. etli* CE3 (wild-type, *n* = 217) and cKD24 (without cumate induction, *n* = 194) cells was followed over a time course of at most 10 h. To minimize damage to the cells during incubation, a system that reduces evaporation (FluoroDish™ plates) was used. Figure 2 shows the type of growth patterns detected, with a summary of the results of cell fate for each strain (Figure 3). As reported for *A. tumefaciens* (Cameron et al., 2015) and *S. meliloti* (Krol et al., 2021), *R. etli* CE3 displays an unipolar division pattern (Figure 2), where deposition of new material is concentrated in the new poles (Supplementary Movie 1). During these time-lapse experiments, no round cells were observed for the wild type strain. In contrast, cells from strain cKD24 showed alterations in several aspects of cell division, including cell division time, polarity and shape (Supplementary Movies 2–4). Since populations of cKD24 are nearly evenly split in two shape classes (round and bacillary) the progeny of each class was followed separately. For mother cells with bacillary shape, most of their progeny was composed of bacillary cells (70.9%), showing unipolar and asymmetric cell growth (Figure 2, row cKD24 rod-rod; Figure 3B), although their cell division time was slower (3 vs. 2 h) than in the wild type cells. Of these bacillary cells, 12.3% had a polarity change (Figure 2, see row cKD24 polarity change), namely, they begin to synthesize cell envelope by the old pole, while the new pole resulted inactivated for growth (Supplementary Movie 4). This change of polarity is a transient effect; further divisions in these cells resume growth from the new poles. A large fraction of mother cells of bacillary shape (28.1%) produced daughter cells with round shape (Figure 2, see row cKD24 rod-round) with a cell division time of 2.5 h. Finally, some of the mother cells of bacillary shape (1%) produced round daughter cells with multiple growth foci, giving a branched appearance.

On the other hand, cell division in mother cells with round shape produced daughter cells with round (62.6%), bacillary (34.1%) and branched (3.3%) shapes. Notably, the bacillary cells that came from mother cells with a round shape, showed polarity change in a higher proportion (29%) than the one seen in daughter bacillary cells from a mother with bacillary shape (12.3%) (Figures 3B,C). Even in these cases the change of polarity is a transient effect, with cells resuming growth from newly-generated poles. Most of the round cells that produced bacillary daughter cells did so solely from a single point in the round cell (Figure 2 see column cKD24 round-rod). Round cells were able to divide and produced new round cells, but they did so with a long division time, of about 8 h (Figure 2 see row cKD24 round-round, Supplementary Movie 2).

A significant proportion of both bacillary (1%) and round cells (3.3%) from cKD24 produced growth foci at several points in the cell, without producing novel cells, giving a branched shape (Figure 2, see row cKD24 round-branch, Supplementary Movie 3). These branched cells remained in this state for the whole course of the experiment, failing to produce new cells. We were unable to detect a growth pattern, either, for the type of shape generated by each mother cell, or in the position and number of growth sectors, an effect that is clearly seen in the round cells that grow from several points of the cell in a quasi-simultaneous manner (Supplementary Movie 3). There are reports in Rhizobiales of null mutants that generate round cells (Kobayashi et al., 2009; Calatrava-Morales et al., 2017; Zupan et al., 2019; Krol et al., 2020). What is unusual in this case is that depletion of RdsA produced a wealth of effects, modifying cell morphology, polarity, and time and number of growth foci. All these point toward a major disruption of the cell division program in these cells.

### Depletion of RdsA Causes a Global Change in Gene Expression

To identify which genes presented alterations in expression in strain cKD24, we undertook a RNAseq analysis using the Illumina system (see section “Materials and Methods”). For that, both *R. etli* strains CE3 and cKD24 were grown in minimal medium (without cumate induction) to mid-exponential phase. Three biological replicates were used for each strain. Details for this analysis are shown in Supplementary Datasets 1C–E and Supplementary Figure 3.

**FIGURE 4:**
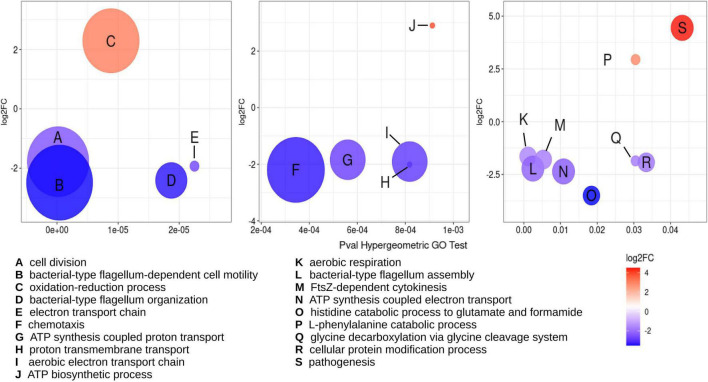
Multiple biological processes are overrepresented among DEGs detected under RdsA depletion. Hypergeometric analysis was used for determination of biological processes overrepresented in the DEGs for the cKD24 mutant, using a *p*-value cutoff for the hypergeometric GO test ≤ 0.05. Overrepresented GO terms are shown with a letter inside each circle (see legend at the bottom). Circle diameter is proportional to the number of DEGs grouped in a GO term. The upregulated and downregulated DEGs are shown in red and blue circles, respectively, with a cutoff threshold of log2FC ≥ 1.5 and P-adj/FDR ≤ 0.02.

Using as criteria a FDR or adjusted *p*-value cutoff ≤ 0.02 and a log2 fold change of 1.5, a total of 1,105 genes were classified as differentially expressed genes (DEGs). Of these, 437 DEGs were downregulated, while 668 DEGs were upregulated (Supplementary Dataset 1E). Functional classification of the DEGs was achieved using the Gene Ontology (GO) database by biological processes. Assignation to GO groups belonging to clear biological processes was possible only for 352 DEGs, indicating that most of the changes in expression occur in genes without a known function. To evaluate if there are significant overrepresentation of particular biological processes among the functionally annotated DEGs, these were subjected to a hypergeometric analysis (Figure 4). From this analysis, 101 DEGs were identified as belonging to significantly overrepresented groups. The most overrepresented groups were cell division (including FtsZ-cytokinesis), oxidative respiration (comprised of electron transport chain, ATP synthesis-coupled proton and electron transport, proton transmembrane transport, aerobic electron transport chain, and aerobic respiration), and cell motility (including chemotaxis, flagellum-dependent cell motility, and organization and assembly of the flagellum). The DEGs belonging to these three biological processes were mostly downregulated. On the other hand, the most prominent, overrepresented biological processes that were found to be upregulated are the oxidation-reduction process and pathogenesis (Figure 4). The subset of DEGs comprising the oxidation-reduction process is comprised of proteins with a general prediction of oxidoreductases. No genes that participate in tolerance to oxidative stress were found among the DEGs. The pathogenesis group is composed of proteins belonging to the BA14K protein family, which are poorly characterized.

**FIGURE 5:**
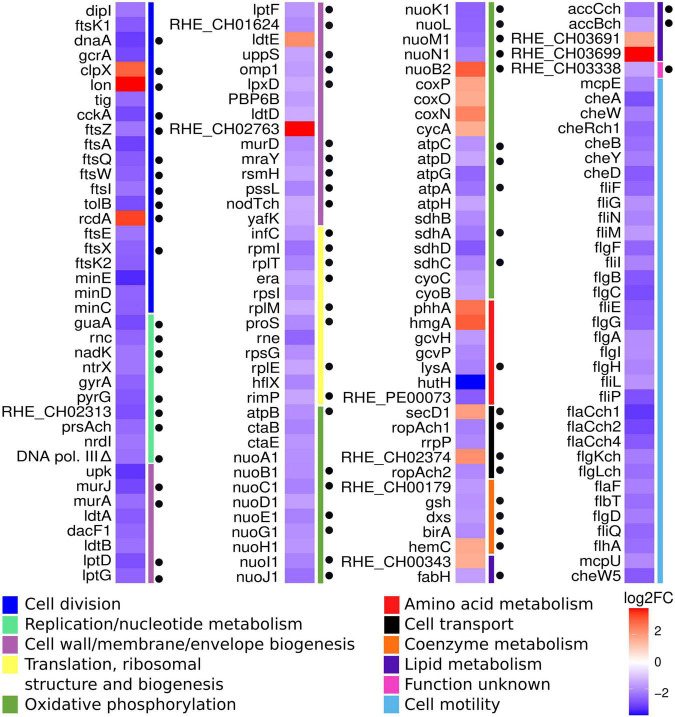
DEGs detected under RdsA depletion correspond to possible essential genes. Heatmap showing significant DEGs in the cKD24 mutant, grouped according to their function (colored bars at the right of the map), based on the COG database (see legend at the bottom). Red, upregulated DEGs; blue, downregulated DEGs (cutoff threshold of log2FC ≥ 1.5 and P-adj/FDR ≤ 0.02). Black circles located on the right side of the heatmap mark orthologs to essential genes of *A. tumefaciens*.

This analysis was complemented by classification of all the DEGs according to their function in the COG (Clusters of Orthologous Groups) database (Figure 5). This approach revealed groups of genes that correspond in function to those identified by hypergeometric analysis, such as cell division, oxidative phosphorylation and cell motility. Interestingly, 55 DEGs belonging to novel groups were easily detected, corresponding to the classes of replication/nucleotide metabolism; cell wall/membrane/envelope biogenesis; and translation, ribosomal structure, and biogenesis. This combined approach allowed the establishment of a collection of 156 DEGs to be analyzed (Figure 5). Many of these DEGs appear in classes that may be considered essential for bacterial growth. For that reason, we carefully reviewed the existence of orthologs to essential genes among the DEGs. Currently, there is no report about essential genes in *R. etli*, so the list of DEGs was examined looking for orthologs to essential genes of *A. tumefaciens* (Curtis and Brun, 2014). In this way, we were able to establish the existence of 70 potentially essential DEGs in cKD24 (Figure 5).

### RdsA Depletion Causes a Decrease in Expression of Essential Genes for Cell Division and Cell Wall Biogenesis

**FIGURE 6:**
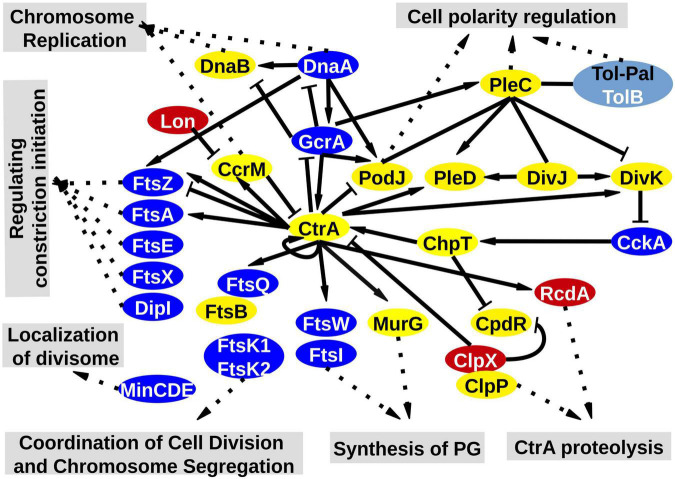
cKD24 mutant showed DEGs in the canonical cell division pathway for alphaproteobacteria. DEGs in the cKD24 mutant that were downregulated (blue ovals) or upregulated (red ovals) with a cutoff threshold of log2FC ≥ 1.5 and P-adj/FDR ≤ 0.02. Non-DEGs are shown in yellow ovals. TolB (in white letters) was the only downregulated gene in the Tol-Pal complex (light blue oval). The cell division pathway was based on the KEGG pathway map for Cell cycle in *C. crescentus.*

As expected from the phenotype exhibited by the cKD24 mutant, significant alterations were found in 21 genes participating in cell division. Most of these changes were corroborated by RT-qPCR analyses (Supplementary Figure 4). These are depicted in the KEGG map corresponding to cell division, based on the cell division pathway of *C. crescentus* (Laub et al., 2002), as shown in Figure 6. Most of these genes are conserved in Rhizobiales, such as *S. meliloti* (Pini et al., 2015). The first aspect to highlight is that the genes *ftsA, E, I, K1, K2, Q, W, X*, and *Z*, whose products comprise most of the divisome, as well as the *minCDE* operon, participating in the correct location of the septum (Rothfield et al., 2005), were downregulated. Significant reductions in expression were also seen for *dnaA* (responsible for genome replication in coordination with cell division), *dipI* [a periplasmic protein that interacts with the *ftsQLB* complex (Osorio et al., 2017)], *gcrA* (an activator of *ctrA*) and *cckA* (a cell-cycle kinase). Interestingly, the master cell division regulator CtrA (Mann et al., 2016) was not downregulated (Figure 6 and Supplementary Figure 4). On the other hand, genes such as *pleC* (Kim et al., 2013), *podZ* (Grangeon et al., 2017; Howell et al., 2017), *popJ* (Fields et al., 2012; Anderson-Furgeson et al., 2016), *divK, divJ* (Pini et al., 2013), and GPR (Zupan et al., 2019, 2021), participating in determination of asymmetric and unipolar growth, displayed normal expression levels in the cKD24 mutant. The downregulation in many genes for cell division suggests a major perturbation in cell division, which is consistent with the longer cell division times and cell division problems exhibited by the cKD24 mutant (Figures 2, 3 and Supplementary Movies 2–4). These data indicate that depletion of RdsA affects, either directly or indirectly, a sizable fraction of genes involved in cell division.

**FIGURE 7:**
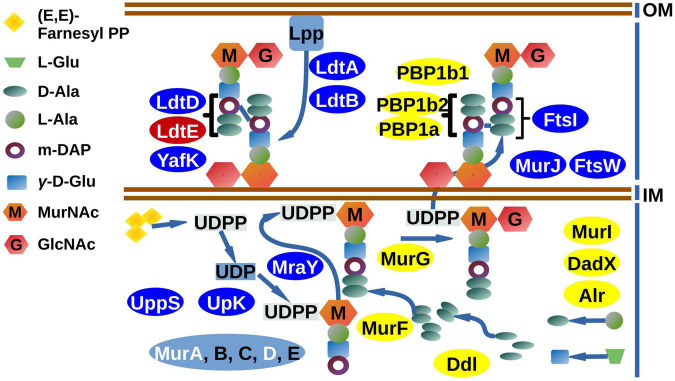
DEGs are part of the canonical cell wall biogenesis pathway. DEGs in the cKD24 mutant that were downregulated (blue ovals) or upregulated (red ovals) with a cutoff threshold of log2FC ≥ 1.5 and P-adj/FDR ≤ 0.02. Non-DEGs are shown in yellow ovals. DEGs downregulated in the *murA-murE* gene cluster (light blue oval) are shown in white letters. The pathway map is based on KEGG Pathway Maps: Peptidoglycan biosynthesis, D-Alanine, and D-glutamate metabolism.

For the PG synthesis pathway (Figure 7), extensive downregulation, verified by RT-qPCR analysis (Supplementary Figure 4) was also detected. The *uppS* and *upK* genes, performing the synthesis of di-trans, poly-*cis*-undecaprenyl phosphate from farnesyl diphosphate, were shown to be downregulated. Downstream in the pathway, the genes *murA, murE*, and *mraY* (involved in the synthesis of precursors of PG) displayed reduced levels in the cKD24 mutant. Reductions in the expression levels of both *ddl* and *alr* genes, which participate in synthesis of D-amino acids, were undetectable in the RNAseq analysis, but showed a distinct downregulation in the RT-qPCR analysis. Low gene expression was also seen for genes, such as *murJ*, participating apparently in flipping of the PG precursors across the plasma membrane for subsequent polymerization (Egan et al., 2020) and *ftsW*, a peptidoglycan glycosyltransferase (Taguchi et al., 2019). These observations point toward a general decrease in the synthesis of PG precursors.

Furthermore, the penicillin-binding proteins (PBP) *ftsI* and PBP1b1, which are responsible for the transpeptidation and transglycolyzation of PG molecules during the cell division cycle (Cameron et al., 2014), were downregulated in the cKD24 mutant, although the reduction in expression of PBP1b1 was only detected in the RT-qPCR analysis (Supplementary Figure 4). Interestingly, similar levels of expression to the wild-type cells were seen for PBPs that participate in cell elongation, such as PBP1c, or essential for polar growth in *A. tumefaciens* and *S. meliloti*, as PBP1a (Williams et al., 2021). Down regulation was also observed for low molecular weight PBPs, such as PBP6B and *dacF*. The significance of these reductions is difficult to ascertain, given that its individual participation might be dispensable for the polymerization of PG, due to their redundant activities (Egan et al., 2020; Williams et al., 2021). However, cell branching and ectopic cell poles have been reported for *E. coli* cells lacking multiple low molecular weight PBPs (Nilsen et al., 2004; Potluri et al., 2012). For the LD-transpeptidases, participating in PG biosynthesis during cell elongation (Egan et al., 2020; Aliashkevich and Cava, 2021), enhanced expression was found for *ldtE*, but lower expression was seen for *ldtA*, *ldtB*, and *ldtD*. Interestingly, one of these (*ldtB*) localizes to the growth pole, at least in *A. tumefaciens* (Cameron et al., 2014; Grangeon et al., 2015). These data indicate that RdsA may function as an activator, either directly or indirectly, of the PG biosynthetic pathway. The complex phenotype displayed by mutant cKD24, characterized by a change in cell shape and altered cell division, may be the product of the reductions in expression of genes for cell division and PG biosynthesis.

### RdsA Depletion Generates Changes in the Expression of Essential Genes in Oxidative Respiration and Translation

Concerning the DEGs involved in oxidative respiration, most of the genes of the *nuo* clusters, which encode respiratory complex I, were shown to be downregulated. Similar behavior was observed for the succinate dehydrogenase operon (*sdhBADC*), comprising respiratory complex II. A large fraction of the genes that encode the ATP synthase complex in *R. etli*, affecting both the F_1_ (*atpA, C, D, G, H*) and the F_0_ (*atpB*) regions were also downregulated in cKD24. The marked reduction in expression of genes for respiratory complexes suggests that the mutant cKD24 could face difficulties in the generation of energy.

Surprisingly, reductions in expression of several genes that participate in ribosome structure and function, corroborated by RT-qPCR (Supplementary Figure 4), were also seen. For structural ribosomal proteins, S7 (*rpsG*), L5 (*rplE*), L13 (*rplM*) and L35 (*rpmI*) displayed reduced expression in cKD24. Significant reductions were also seen for *infC* that encodes for the translation initiation factor IF-3 (Singh et al., 2005) and *proS* encoding the proline-tRNA ligase (Zajac et al., 2020). These results suggest that cKD24 may present problems in the synthesis of proteins.

## Discussion

In this study we show that *rdsA* plays a crucial role in determining cell division and shape in *Rhizobium etli*. Depletion of RdsA causes a round cell phenotype, longer generation time and changes in cell polarity, causing some of the cells to start growing from the old pole. Ectopic growth poles were also seen, giving rise to branched cells. It should be stressed that these changes were seen upon long-term depletion of RdsA. Due to technical limitations, we were unable, at this time, to institute a short-term depletion regime. Thus, it should be kept in mind that an unknown fraction of the changes that we see might be indirect or pleiotropic. Despite this caveat, the combination of changes observed is unusual among mutants affecting cell division in Rhizobiales. Regarding cell shape, round cells were detected upon depletion of GPR in *A. tumefaciens* (Zupan et al., 2019, 2021) or its ortholog (*rgsE*) in *S. meliloti* 1021 (Krol et al., 2020), as well as upon elimination of *cpdR* in *S. meliloti* 1021 (Kobayashi et al., 2009) or *ntrY* in *S. meliloti* GR4 (Calatrava-Morales et al., 2017). A round cell phenotype has been described also in cells that overproduce RgsM in *S. meliloti* 1021 (Schäper et al., 2018). Interestingly, branched cells or changes of polarity were not described in these cases, and no changes in the expression for any of these genes were found in cKD24. Changes in stoichiometry of specific proteins in the *minCDE* system of *S. meliloti* 1021 may affect cell shape and branching (Cheng et al., 2007). These effects require imbalance between the *min* components, such as elimination of *minE* but preservation of the other proteins, and were not seen upon knocking-out of the whole operon. Overproduction of *minCDE* produces filamentous cells, with multiple branching points (Cheng et al., 2007). In contrast, reduced expression of the *minCDE* was observed in the cKD24 mutant. Our previous work (Landeta et al., 2011) revealed that a mutation in *minCDE* produced a high amount of minicells.

Medium-dependent shape changes toward round cells, sometimes coupled with delocalization of a polar protein, were seen upon overproduction of specific proteins in *S. meliloti*, such as RgsA, RgsB, RgsC or RgsS, but not upon their depletion (Krol et al., 2020). Moreover, expression of the corresponding orthologous genes remained unaltered in mutant cKD24.

Formation of ectopic growth poles, branching and changes in polarity were seen upon depletion of polar-organizing proteins or global regulators. For instance, deletion of regulators such as *divK*, *pleC* or the *pleC*-related regulator *pdhS1* in *A. tumefaciens* generated branched cells with ectopic growth poles (Kim et al., 2013). In *A. tumefaciens*, either elimination or depletion of *popZ* lead to the formation of ectopic growth poles, branching and even minicell formation (Grangeon et al., 2017; Howell et al., 2017). Mutants depleted of PodJ in *A. tumefaciens* revealed a high proportion of branched cells with ectopic growth poles, as well as a low proportion of minicells (Anderson-Furgeson et al., 2016). In these mutants, a high proportion of cells with a changed polarity (as much as 47%), were also seen, linked to a mislocalization of the PopZ protein (Anderson-Furgeson et al., 2016). Similarly, a *podJ1* mutant in *S. meliloti* showed a medium-dependent apparition of branched cells, sometimes with multiple growth poles (Fields et al., 2012). Alterations in the expression of these genes were not seen for mutant cKD24. In fact, the combination of effects seen upon depletion of RdsA (round cell shape, changes in polarity, ectopic growth poles and branched cells), remains unique among mutants affecting cell division in the Rhizobiales.

The transcriptomic analysis of mutant cKD24 reveals that RdsA acts as a global transcriptional regulator. Although many of the genes with altered expression in the mutant have not been characterized, it is clear that depletion of RdsA alters, either directly or indirectly, at least five biological processes, namely cell division, cell wall biosynthesis, aerobic respiration, translation and motility. Regarding cell division, reduced transcript levels were detected for many of the genes that participate in divisome assembly (such as *ftsA, E, I, K1, K2, Q, W, X*, and *Z*) and proper septum formation (*minCDE*). Reduced transcript levels were also seen for *dnaA* (crucial for DNA replication) *dipI* (interacting with *ftsQLB*) and for global regulators such as *cckA* and *gcrA*, but not *ctrA*. For cell wall biosynthesis, reduced transcript levels were seen for genes participating in almost every step of peptidoglycan biosynthesis, including precursor synthesis, flipping, transpeptidation and transglycolyzation. Aerobic respiration also showed extensive downregulation, affecting complex I (the *nuo* clusters), complex II (succinate dehydrogenase system), and complex V (ATP synthase). Genes encoding proteins of significance for the translation process were also downregulated, including four ribosomal proteins (for the small and large subunits of the ribosome), an initiation factor for translation (IF-3) and a unique gene for tRNA_*pro*_ synthesis (*proS*). Cell motility was also affected, with significantly reduced expression in 36 out of the 52 genes that comprise the motility cluster.

It is not easy to explain the pattern of expression displayed by mutant cKD24 by invoking control of known regulatory genes involved in each biological process. For cell cycle control, the master regulatory gene in many bacteria is *ctrA*, but *ctrA* was not differentially expressed in cKD24. However, alterations in functioning of the CtrA system may occur at the posttranscriptional level, which it was not explored in this work. Although the *ctrA* regulon has not been described in *R. etli*, it was reported for the related bacterium *S. meliloti* (Pini et al., 2015). An inspection of the differentially expressed genes in the *ctrA* depleted strain of *S. meliloti* and cKD24 in *R. etli*, revealed just a few relevant similarities, restricted mainly to *dipI*, *mcpA*, *mcpE*, *mcpZ*, *minCDE*, and PBP6b. Similarly, transcriptomic analysis of strains depleted or lacking known regulatory genes involved in cell division, such as *ntrY* in *S. meliloti* GR4 (Calatrava-Morales et al., 2017), *cdnL* (Woldemeskel et al., 2020) and *hfq* (Irnov et al., 2017) in *C. crescentus*, did not reveal similarities with the transcriptional pattern described for the depletion of RdsA.

The phenotypic effects of depletion of RdsA on cell shape, division and polarity can be attributed to the changes in functioning of key genes for cell division and peptidoglycan assembly. At this moment, is not possible to ascertain what components are under the direct control of RdsA, and which are due to indirect control. The main difficulty is that RdsA is a predicted hybrid histidine kinase, but its response element remains unknown, a point which will be the subject of future work. As mentioned before, convincing orthologs to *rdsA* are mainly restricted to the Rhizobiales, including Agrobacterium and Rhizobium species.

Coordination between division and cell metabolism is an essential aspect for the evolutionary success of any organism (Sperber and Herman, 2017; Bergé et al., 2020). RdsA in Rhizobium may be a key regulator controlling a plethora of functions relevant not only to division, but also for general cell metabolism. Understanding this mechanism, including the target genes subject to direct control, will be the aim of future work.

## Materials and Methods

### Growth Conditions

All the *Rhizobium etli* strains were grown in PY medium supplemented with calcium chloride (Noel et al., 1984) or in MMY minimal medium (Bravo and Mora, 1988) supplemented with biotin at 1 mg l^–1^, at 30°C. When needed, cumate (10 μg ml^–1^) or Isopropyl β-D-1-thiogalactopyranoside (IPTG, 1.0 mM) were added as inducers. *Escherichia coli* strains were grown in LB broth at 37°C. When needed, antibiotics were added at the following concentrations (in μg ml^–1^): kanamycin (Kan), 30; nalidixic acid (Nal), 20; gentamicin (Gm), 30 and spectinomycin (Sp), 100, tetracycline (Tet), 10. For *R. etli* growth curves, overnight cultures of the appropriate strains in PY medium were centrifuged, washed in saline solution and used to inoculate 200 μl microplate cultures to an A_600_ of 0.05. Microplates were incubated at 30°C, with hourly determinations of absorbance in a BioTek Synergy™HT reader. For viability determinations, the alamarBlue™ HS cell viability reagent (Invitrogen) was used, according to the instructions of the manufacturer.

### Primers and Strain Construction

The conditional knockdown (cKD) mutagenesis employed aimed to put gene *rdsA* (RHE_PE00024) under the control of a cumate-inducible promoter. To that end, a region encompassing the transcriptional repressor CymR in conjunction with the PR/cmtO promoter (Chubiz et al., 2013) was amplified by PCR and ligated into pK18mob (Schäfer et al., 1994), generating pMASQ plasmid. The specific knockdown vector was generated by amplification of a 306 pb segment of *rdsA* (encompassing the translation initiation site and 295 bp downstream) and ligated into pMASQ, giving rise to pMASQ*:rdsA*_300nt_. This plasmid was transformed into *E. coli* S17-1 and used as donor in plate biparental matings with *R. etli* CE3, selecting for transconjugants resistant to Nal and Kan. A single-crossover recombination event with the homologous target cointegrates the whole plasmid, producing the displacement of the native *rdsA* promoter and the incorporation of the cumate-inducible promoter upstream of the full-length *rdsA* gene (strain cKD24). To minimize apparition of second-site suppressors of cKD24, all experiments were started either from newly-constructed cKD24 or from frozen stocks preserved in glycerol. For complementation, the full-length *rdsA* was cloned into the replicative plasmid pSRKGm (Khan et al., 2008), under the control of a *lac*-inducible promoter, with the restriction enzymes *Nde*I-*Bam*HI, giving rise to pSRKGm*:rdsA*. Primers employed in this work are shown in Supplementary Datasets 1A,B. All primers were designed with Primer-BLAST software, and purchased from commercial providers. Taq DNA polymerase (Thermo Scientific) and Platinum Taq polymerase (Invitrogen), were used in PCRs.

### Microscopy

For static visualization, samples of 9 h liquid cultures in PY medium (either with or without inducer) of *R. etli* strains were spotted on agarose beds (1% in PBS buffer, Grace Bio-Labs SecureSeal™ imaging spacer) and analyzed on a Nikon Eclipse Ti inverted microscope, using 100X/1.45 NA Plan Apochromat oil objective. Images were recorded with a Nikon DS-Qi2 camera. For DAPI (4′,6-diamidino-2-phenylindole) staining, cell pellets (washed thrice with PBS buffer), were resuspended in PBS and DAPI was added to a final concentration of 3 μg ml^–1^, cells were incubated in the dark for 5–10 min at ambient temperature. Cell washed three times in the same PBS buffer were spotted on an agarose pad and observed. Fluorescence was detected using a maximum emission of 456 nm, excitation of 340 nm filters. For time-lapse microscopy, samples of 9 h liquid cultures in PY medium of *R. etli* strains were spotted on agarose pads (1% in MMY medium, FluoroDish™ plates). Images were captured every 10 min for 24 h with Nikon Eclipse Ti inverted stand, using 100X/1.45 NA Plan Apochromat oil objective, a Nikon DS-Qi2 camera and the Nikon’s Digital Sight DS-U3 camera controller. Control of imaging parameters was done with Nikon NIS-Elements AR 4.20 software and a perfect focus system. Temperature (30°C) and humidity control was maintained with a Lexan Enclosure Unit with Oko-touch temperature control. Time-lapse series were analyzed with Fiji (Schindelin et al., 2012).

### Bacterial Two-Hybrid Homodimerization Assay

A LexA-based two-hybrid system (Dmitrova et al., 1998) was used. Full-length *rdsA* gene was amplified by PCR from genomic DNA of *R. etli* CE3, ligated into the LexA plasmid (pSR658-A) and transformed into *E. coli* SU101. *E. coli* SU101 harboring different plasmid constructs were grown (A_600_ = 1.0) in LB medium with Tet, Kan, and IPTG. The β-galactosidase and microplate protein concentration determinations were as previously described (Guadarrama et al., 2014). The extent in reduction of β-galactosidase activity was used as a proxy of homodimerization efficiency (Dmitrova et al., 1998). For complementation with *rdsA*W169R in *R. etli*, the wild type *rdsA* gene was excised from plasmid pSRKGm*:rdsA* and substituted by a *Blp*I-*Aat*II segment harboring *rdsA*W169R (generated by PCR, Supplementary Dataset 1A). pSRKGm*:rdsA* and pSRKGm*:rdsA*W169R were transferred separately to cKD24 by biparental matings, selecting for transconjugants on PY medium with Kan, Nal, and Gm.

### Western Blotting

Samples were subjected to SDS-PAGE (12% polyacrylamide) and transferred to 0.45 μm PVDF Immobilon-P membranes (Millipore) in a Tank transfer system (Bio-Rad). Membranes were blocked with 5% non-fat milk, incubated with anti-LexA polyclonal antibody or anti-DnaK monoclonal antibody (Ab-cam) and washed with PBS 1X, 0.05% Tween 20. Immunodetection was performed with a 1:15,000 dilution of horseradish peroxidase-conjugated anti-rabbit (polyclonal) or anti-mouse (monoclonal) antibodies (MDL Millipore Corporation) and visualized with an Immobilon Western chemiluminescence reagent kit (Millipore).

### RNAseq Analysis

Uninduced *R. etli* strains were grown in MMY at 30°C to an *A*_620_ of 0.5 (9 h). Three biological replicates were set up for each strain analyzed. Cell pellets for each replicate (10^8^ cells) were stored on RNAlater (Thermo-Fisher). RNA extraction, rRNA depletion, library preparation and Illumina sequencing was done by GeneWiz (South Plainfield, NJ, United States). Quality checking was done using FastQC^1^ and sequences were trimmed using Trimmomatic v.0.36 (Bolger et al., 2014). Paired-end reads were mapped toward *R. etli* CFN42 reference genome (GenBank accession number GCF_000092045.1) using Bowtie2 aligner v.2.2.6. (Langmead and Salzberg, 2012). Read counts for each CDS (6226) were generated using featureCounts software (Liao et al., 2014), allowing MultiMapping and MultiOverlap reads. For Principal Component Analysis (PCA), the NOISeq PCA function (Tarazona et al., 2015) was used, and clustering was done with Distance Matrix Computation package in R using the function dist^2^, with normalized counts in natural logarithm. Heatmaps were visualized using pheatmap^3^ of R. Differential expression analysis was done with NOISeqBio (Tarazona et al., 2015). Reads were filtered with NOISeq using method 1 and cpm = 2. Data post filtering were used for differential gene expression in NOISeqBIO, using TMM normalization. For identification of significantly Differentially Expressed Genes (DEGs), False Discovery Rate (FDR) or adjusted *p*-value cutoff ≤ 0.02 and log2 fold change ≥ 1.5 were used. Categorization of DEGs was achieved using the Clusters of Orthologous Groups database (Galperin et al., 2021), and mapped to known metabolic pathways with KEGG Mapper (Kanehisa and Sato, 2020). Significant overrepresentation of gene ontology (GO) groups among DEGs was determined by hypergeometric testing with Gostats (Falcon and Gentleman, 2007), using a *p*-value cutoff ≤ 0.05. The universe used was GO terms in the *R. etli* genome, recovered from Uniprot (The UniProt Consortium, 2017), with the GO database of Gostasts. For evaluation the essential genes in the DEG dataset, entries corresponding to *Agrobacterium tumefaciens* str. C58 in the Database of Essential Genes [DEG (Luo et al., 2014)] were scanned. Matches in the DEG dataset were verified by BLASTP bidirectional best-hit analysis.

### Quantitative PCR Analysis

Transcriptional differences were validated by reverse-transcription quantitative PCR (RT-qPCR) analysis. All primers (Supplementary Dataset 1B) amplify 100–300 bp regions. RNA was isolated using Trizol Reagent (Zymo Research), and DNA traces were removed by DNAase I treatment. Absence of DNA from RNA samples was corroborated by PCR with *rpoA* gene primers, and RNA integrity was verified by gel electrophoresis. RNA Purity and concentration were quantified with Nanodrop 20000 Spectrophotometer (Thermo Scientific™). cDNA was generated with 2.5 μg of RNA, using RevertAid™ H Minus First Strand cDNA Synthesis kit (Thermo Scientific™). RT-qPCR analysis was carried out on a StepOnePlus (Applied Biosystems™) Real-Time PCR System using Maxima SYBR Green/ROX qPCR Master Mix (Thermo Scientific) according to the manufacturer instructions. Three biological replicates were performed for each strain/condition, using three technical repeats. Expression levels were normalized using *rpoA* as reference gene (Supplementary Dataset 1B). The relative expression (RQ) was determined as 2^ΔΔCT^. To detect significant differences between samples, an unpaired Student’s *t*-test (*t* test function of the stats package in R) was used, employing the RQ values and a cutoff *P* ≤ 0.05. RQ values were converted to log2 fold changes, where RQ values ≥ 0.65 are shown as positive log2 fold change, and RQ values ≤ 0.65 as negative log2 fold change. To give a negative value of RQ values ≤ 0.65, we use the formula (-1)/RQ.

## Data Availability Statement

The RNAseq data generated in this study have been deposited in the National Center for Biotechnology Information Gene Expression Omnibus database, https://www.ncbi.nlm.nih.gov/geo/ (GEO Series accession no. GSE184428).

## Author Contributions

DR conceived the study through discussion with all the co-authors, supervised the work, got the funding, and, together with SM-A, wrote the draft manuscript. SM-A constructed the knockdown mutant, carried out all the microscopical visualization and time-lapse experiments, and performed the bioinformatic analysis of the RNAseq experiment. CG performed the experiments for homodimerization analysis and constructed complemented strains. AD lent general lab support and performed initial work for characterization of the rdsA gene. All authors contributed to the article and approved the submitted version.

## Conflict of Interest

The authors declare that the research was conducted in the absence of any commercial or financial relationships that could be construed as a potential conflict of interest.

## Publisher’s Note

All claims expressed in this article are solely those of the authors and do not necessarily represent those of their affiliated organizations, or those of the publisher, the editors and the reviewers. Any product that may be evaluated in this article, or claim that may be made by its manufacturer, is not guaranteed or endorsed by the publisher.

## Footnotes

1
https://www.bioinformatics.babraham.ac.uk/projects/fastqc/

2
https://cran.r-project.org/web/packages/parallelDist/index.html

3
https://CRAN.R-project.org/package=pheatmap
